# T cell immunotherapy for solid tumors: limitations, progress, and future prospects

**DOI:** 10.3389/fimmu.2026.1755751

**Published:** 2026-01-29

**Authors:** Yinuo Wang, Boyu Zhang, Yajie Wang, Yuan Tan, Xiaoqian Hu, Xuan Che, Mei Feng

**Affiliations:** 1 College of Life and Environment Science, Huangshan University, Huangshan, Anhui, China; 2 Department of Immunology, School of Basic Medical Sciences, Peking University Health Science Center, Beijing, China; 3 College of Engineering, University of California at Berkeley, Berkeley, CA, United States; 4 Department of Gastrointestinal Surgery, Peking University First Hospital, Beijing, China; 5 Department of Clinical Laboratory, Zhejiang Cancer Hospital, Hangzhou, Zhejiang, China; 6 Department of Cell biology, School of Basic Medical Sciences, Peking University Health Science Center, Beijing, China

**Keywords:** chimeric antigen receptor T cells, immunotherapy, T cell receptor-engineered T cells, T cell therapy, tumor microenvironment

## Abstract

T cell-based immunotherapies have achieved notable success in the treatment of hematological malignancies, particularly through the application of chimeric antigen receptor (CAR) T cells. However, the clinical efficacy of such approaches in solid tumors remains limited due to a range of intrinsic and extrinsic barriers, including tumor antigen heterogeneity, the immunosuppressive tumor microenvironment (TME), and insufficient T cell infiltration and persistence. Despite these challenges, significant advances have been made in recent years in the development of CAR-T cells, T cell receptor-engineered T cells (TCR-T), and tumor-infiltrating lymphocytes (TILs) for solid tumors. This review provides a comprehensive overview of the current landscape of T cell immunotherapies targeting solid tumors. We examine the underlying mechanisms and design principles of each therapeutic modality and summarize the clinical progress in a tumor-specific context. Particular attention is given to the biological and technical challenges that impede treatment efficacy, including antigen escape, on-target off-tumor toxicity, and the suppressive features of the TME. Furthermore, we discuss emerging strategies aimed at overcoming these obstacles, such as combinatorial antigen targeting, immune checkpoint blockade, synthetic biology tools, and gene editing technologies. Finally, we outline future perspectives in the field, emphasizing the importance of precision immunotherapy and the integration of multi-omics data to enhance T cell functionality and specificity. This review aims to inform ongoing research and guide the clinical translation of T cell-based therapies for solid tumors.

## Graphical Abstract

**Figure:**
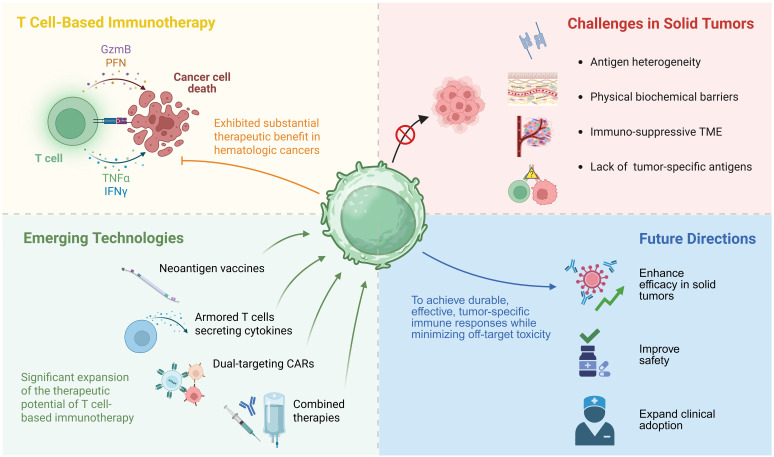

## 1 Introduction

T cell-mediated immunotherapy has emerged as a transformative modality in cancer treatment, particularly with the advent of adoptive cell transfer strategies such as tumor-infiltrating lymphocytes (TILs) (1–3), chimeric antigen receptor (CAR) T cells (4, 5), and T cell receptor-engineered T cells (TCR-T) (6–8). These approaches have demonstrated remarkable therapeutic efficacy in hematologic malignancies, leading to several regulatory approvals and widespread clinical adoption (9). However, their application to solid tumors has been considerably less successful, owing to a multitude of biological and technical challenges that are unique to the solid tumor microenvironment.

Solid tumors are characterized by pronounced antigen heterogeneity, physical and biochemical barriers to T cell infiltration, and an immunosuppressive tumor microenvironment (TME) that actively impairs T cell activation, expansion, and cytotoxic function (10, 11). Moreover, the identification of truly tumor-specific antigens in solid tumors remains a significant hurdle, increasing the risk of on-target off-tumor toxicities when shared antigens are targeted (12). These factors collectively contribute to suboptimal therapeutic responses and pose substantial challenges to the development of safe and effective T cell-based therapies for solid malignancies.

**Figure 1:**
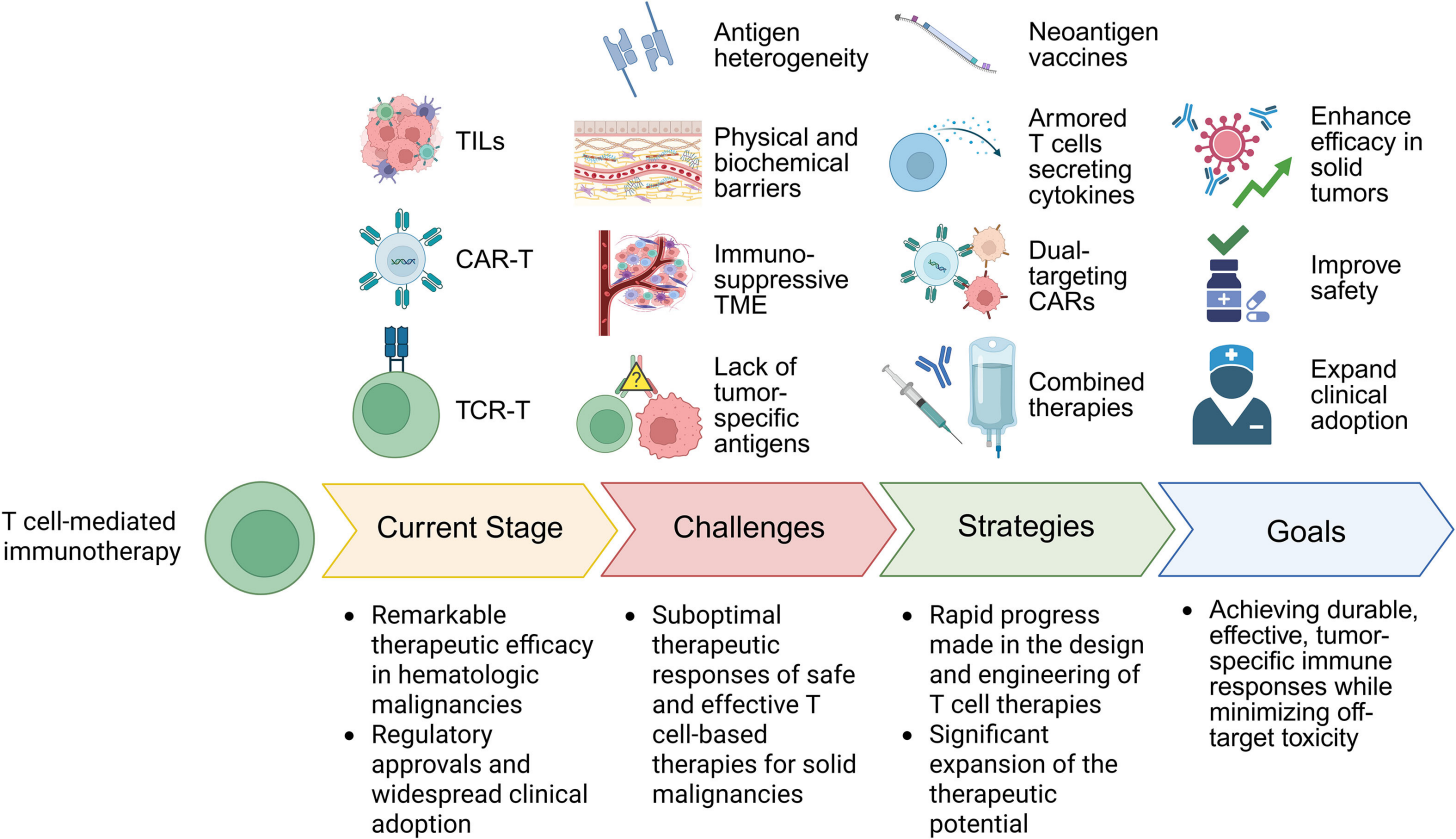
Illustration of the overall structure of this review. This diagram illustrates the current stage of T cell-based immunotherapies, the major challenges limiting their efficacy in solid tumors, the emerging strategies designed to overcome these barriers, and the ultimate therapeutic goals. This figure was created in BioRender.

Despite these limitations, rapid progress has been made in the design and engineering of T cell therapies with enhanced anti-tumor capabilities. Innovations such as neoantigen vaccines, armored T cells secreting cytokines, and dual-targeting CARs have expanded the therapeutic potential of adoptively transferred T cells (13, 14). Concurrently, numerous clinical trials are exploring combinatorial regimens that incorporate checkpoint blockade, cytokine support, or targeted therapies to augment T cell efficacy in solid tumors. Given the expanding body of evidence and ongoing technological innovations, it is imperative to critically evaluate the current landscape of T cell immunotherapy for solid tumors. In this review, we first outline the primary challenges that limit the effectiveness of T cell-based therapies in solid tumors, followed by emerging strategies developed to address these barriers. An updated overview of current clinical applications across different tumor types is also provided. **Figure 1** provides a schematic summary of the overall structure of this review, from challenges to strategies and current applications. By integrating insights from basic science, translational research, and clinical trials, we seek to provide a comprehensive and forward-looking perspective on the development of effective T cell-based immunotherapies for the treatment of solid tumors.

## 2 Major obstacles to the efficacy of T cell immunotherapy in solid tumors

### 2.1 Tumor antigen heterogeneity and the lack of universal tumor-specific targets

One of the major challenges in the application of T cell-based immunotherapies to solid tumors is the pronounced intra- and inter-tumoral heterogeneity (15–17). Hematologic malignancies and solid tumors differ markedly in both the degree and nature of heterogeneity. The success of CD19 CAR-T therapy in B-cell lymphoma is not solely due to relatively uniform antigen expression, as CD19 is not a tumor-specific antigen, but rather reflects the clinical tolerability of complete B-cell depletion (18). Critically, this reliance on single, uniformly expressed antigens, a core design assumption of most CAR-T therapies, does not apply to solid tumors, and this misalignment directly contributes to clinical failures of CAR-T in solid tumors (19). In contrast, even when lineage- or tissue-restricted antigens in solid tumors show comparatively homogeneous expression, on-target/off-tumor toxicity affecting essential normal tissues often limits safe targeting, as exemplified by early HER2 CAR-T trials in one colon cancer patient (20). As Chen et al. highlighted, preclinical models commonly use antigen-homogeneous cell lines, masking the real-world challenge of clonal escape due to antigen heterogeneity in human solid tumors, which leads to overestimation of CAR-T efficacy (19). Moreover, solid tumors exhibit substantially higher functional heterogeneity driven by spatial constraints and diverse tumor microenvironmental niches, further complicating effective immune targeting (21–23). This heterogeneity complicates the identification of universally expressed, tumor-specific antigens that can be safely and effectively targeted.

To overcome this challenge, tumor-specific antigens (TSAs), particularly neoantigens arising from somatic mutations, have become central to personalized T cell therapies (24). Unlike shared tumor-associated antigens (TAAs), TSAs are exclusively expressed by tumor cells, enabling precise immune targeting with minimal off-target toxicity. By contrast, TAAs are not exclusive to tumor cells but are abnormally overexpressed in tumors while being weakly expressed in normal tissues (e.g., carcinoembryonic antigen, CEA, in colorectal cancer), which may lead to immune tolerance or potential off-target autoimmune reactions due to their expression in normal cells. TSAs, on the other hand, are derived from tumor-specific genetic abnormalities (e.g., KRAS G12D mutation in pancreatic cancer) and thus evade central immune tolerance, inducing more robust and specific anti-tumor immune responses. T cell responses to TSAs are considered a “final common pathway” of effective cancer immunotherapy (25), forming the conceptual basis for individualized approaches such as TCR-T therapy and neoantigen vaccines (12, 26). Therefore, robust and high-throughput strategies for TSA identification are essential. In recent years, integrative pipelines combining genomic and transcriptomic sequencing with functional validation assays have significantly advanced the discovery of immunogenic neoantigens. For instance, tandem minigene libraries, MHC multimer-based sorting, and single-cell T cell receptor (TCR) profiling have enabled the sensitive detection of neoantigen-reactive T cells (27, 28). These platforms are further enhanced by computational tools that prioritize candidate peptides based on MHC binding, antigen processing, and immunogenicity (29). However, despite the large number of non-synonymous mutations found in tumors, only a small fraction gives rise to genuine immunogenic neoantigens, underscoring the need to integrate multi-omics prediction, immunogenicity scoring, and *in vivo*/*in vitro* validation (30). This scarcity of immunogenic neoantigens further exacerbates the limitation of single-target CAR-T strategies, as even personalized neoantigen-targeted CAR-T may fail to cover all heterogeneous tumor clones. Resources such as pan-cancer driver mutation databases also offer guidance in identifying clinically meaningful TSAs (31).

Moreover, the sources of TSAs are increasingly recognized as diverse. Clonal somatic mutations, shared by all tumor cells, are preferred due to their stability and therapeutic breadth (32). Yet, noncanonical sources such as aberrant translation products and gene fusions greatly expand the neoantigen landscape (33, 34). TSAs derived from intracellular proteins presented by MHC class I pathways are also gaining attention as “cleaner” targets with less risk of on-target, off-tumor toxicity (35). Additionally, the development of neoantigen-specific TCRs is central to personalized T cell therapies. Multiple strategies have been proposed to identify TCRs with high specificity and affinity. One approach leverages clonal expansion within tumors, as Pasetto et al. showed that highly frequent TCR clonotypes in fresh tumor tissue often reflect antigen reactivity, enabling antigen-agnostic discovery (36). Meanwhile, Strønen et al. demonstrated that naïve T cell repertoires from healthy donors can recognize patient-specific neoantigens, offering an alternative source when autologous TILs are dysfunctional or limited (37). Direct TIL screening remains effective. Tan et al. isolated tumor-reactive TCRs from esophageal cancer TILs and confirmed their *in vitro* and *in vivo* function (38). However, as Garcia-Garijo et al. noted, only a small fraction of mutations generate true neoantigens, necessitating integrative pipelines that combine computational prediction, immunogenicity scoring, and functional assays, especially for low–mutational burden tumors (30).

TCR source also affects safety and efficacy. Inderberg and Walchli suggested using wild-type TCRs from long-term responders, particularly CD4^+^MHC-II–restricted clones, to balance potency and safety (39). Ahmadzadeh et al. further showed that tumor-infiltrating regulatory T cells (Tregs) harbor unique neoantigen-specific TCRs, offering underexplored sources. Finally, clinical context matters, as TCR-T may suit advanced disease, while vaccines are more appropriate for early-stage tumors (40). Together, these findings shape a multifaceted framework for rational TCR design. Together, these advances underscore the central role of TSAs in overcoming tumor heterogeneity and tailoring immunotherapy to individual patients. Continued progress in multi-omics profiling, antigen processing models, and receptor optimization will further empower TSA-directed strategies to achieve greater precision, efficacy, and durability in the treatment of solid tumors.

### 2.2 Barriers to T cell trafficking and penetration in the solid tumor microenvironment

The clinical success of T cell–based immunotherapies, including immune checkpoint inhibitors, TCR-T, and CAR-T. critically depends on the efficient infiltration and activation of effector T cells within TME (41). However, many solid tumors exhibit an immune-excluded or “cold” phenotype, characterized by low T cell density and limited access to tumor parenchyma, which compromises therapeutic efficacy (42). Notably, CAR-T engineering has long focused on enhancing effector function (e.g., cytokine secretion, cytotoxicity) while underestimating trafficking and penetration barriers as another key design oversight that drives the gap between CAR-T engineering advances and clinical efficacy in solid tumors (19).

One of the primary barriers to T cell entry is the abnormal structure and function of tumor vasculature. Tumor-associated vessels are often chaotic, immature, and lack hierarchical organization, resulting in poor perfusion, increased interstitial pressure, and erratic delivery of immune cells. Continuous VEGF-driven angiogenesis contributes to this abnormality and promotes the formation of leaky, dilated, and tortuous vessels with disrupted endothelial junctions (43). Furthermore, endothelial cells (ECs) in tumors commonly exhibit a state of “endothelial anergy,” wherein they downregulate adhesion molecules such as VCAM-1 and ICAM-1 and fail to express chemokines like CXCL9 and CXCL10 needed to recruit effector T cells (44). This hyporesponsiveness to inflammatory cues is driven in part by constitutive pro-angiogenic signaling and hypoxia-induced HIF-1α expression (44). However, the role of adhesion molecules can be complex, for instance, in renal cell carcinoma, tumor cells have been shown to escape T cell immunity by paradoxically overexpressing VCAM-1, which normally mediates leukocyte extravasation but, in this context may act to sequester or inhibit infiltrating T cells (45, 46). Strategies that stabilize VE-Cadherin or modulate β-catenin signaling in ECs have been shown to enhance selective T cell transmigration while reducing neutrophil infiltration, suggesting that vascular normalization may selectively benefit antitumor immunity (47). Yet, the clinical benefit of such strategies remains limited because CAR-T design does not integrate trafficking-enhancing modifications (e.g., chemokine receptor engineering) to complement vascular normalization, reflecting a disconnect between engineering priorities and clinical requirements. Beyond vascular obstacles, the extracellular matrix (ECM) serves as a physical barrier that impedes T cell motility and tissue penetration (48). Many tumors exhibit a dense and fibrotic ECM composed primarily of collagen and secreted by activated cancer-associated fibroblasts (CAFs) (49). High collagen density not only blocks T cell infiltration but also modulates immune responses by engaging inhibitory receptors or restricting cytokine diffusion (43, 49). Tumor cells may also form tightly packed nests via increased intercellular adhesion, and lack of lymphatic drainage further isolates the tumor core from immune surveillance.

Chemokine dysregulation further contributes to immune exclusion. CAF-derived CXCL12 can form gradients that sequester T cells in stromal regions, preventing their access to malignant cells (50). Additionally, tumors lacking Batf3-lineage CD103^+^dendritic cells fail to produce CXCL9 and CXCL10, which are essential for recruiting cytotoxic T cells into tumor nests and thereby generating a non-T cell-inflamed microenvironment that resists both endogenous immune responses and adoptive T cell therapies (51). Beyond dendritic cells, tumor-intrinsic activation of the WNT/β-catenin pathway has also been shown to suppress CCL4 expression, which further inhibits Batf3^+^dendritic cell recruitment and impairs initial T cell priming (52). In parallel, tumor-associated macrophages (TAMs) also play a pivotal role in chemokine-mediated immune exclusion. Under hypoxic and fibrotic conditions, TAMs adopt an immunosuppressive phenotype and secrete CCL2 and CCL5, which recruit suppressive myeloid cells and divert effector T cells away from the tumor core (53, 54). Importantly, when defining TAMs based on immunosuppressive functions, emerging evidence suggests that they cannot be adequately explained by a simplistic M1/M2 dichotomy, but rather reflect a spectrum of context-dependent functional states shaped by metabolic stress, spatial localization, and microenvironmental cues (55). Furthermore, TAM-derived VEGF and MMP9 promote aberrant angiogenesis, indirectly disrupting T cell infiltration by contributing to vascular disorganization (56), consistent with recent efforts to redefine TAM functional classification beyond the traditional M1/M2 framework (55). Compounding this chemokine dysfunction, recent evidence from breast cancer models indicates that the migratory deficiency may also be intrinsic to antitumor T cells themselves. Specifically, cytotoxic CD8^+^T cells and Th1 cells were found to exhibit impaired chemotaxis relative to Tregs and Th2 cells, a defect mechanistically linked to IFN–STAT1–driven upregulation of regulator of G protein signaling 1 (RGS1). RGS1 acts to suppress chemokine receptor signaling, thereby limiting effector T cell trafficking to the tumor site. Mechanistically, type I interferons, such as IFN-β, have been shown to robustly induce the expression of regulator of G protein signaling 1 (RGS1), which functions as a negative regulator to inhibit G-protein signaling (57). This IFN–driven upregulation of RGS1 acts to suppress chemokine receptor signaling, thereby limiting effector T cell trafficking to the tumor site. Notably, genetic or pharmacologic targeting of RGS1 restored intratumoral migration and significantly improved therapeutic responses (58). Beyond physical and chemokine barriers, the metabolic deregulation of the TME constitutes a formidable chemical barrier, particularly characterized by acidosis. Tumor cells frequently exhibit elevated aerobic glycolysis, leading to the accumulation of lactate and protons, which creates a low-pH microenvironment. This acidic milieu significantly impairs T cell motility, proliferation, and cytotoxic function, effectively acting as a mechanism of immune evasion (59, 60). A recent study highlighted the critical association between pH regulation and the immunological state of the tumor, demonstrating that bicarbonate transporters (such as SLC4A7) on tumor cells regulate intracellular pH while acidifying the extracellular space, thereby maintaining immunological dormancy and excluding T cell infiltration (61). Strategies targeting these pH-regulating mechanisms may therefore restore a favorable environment for anti-tumor immunity (60).

Multiple non-T cell populations within the TME contribute to immunosuppression and impede effective T cell responses. Tumor-associated natural killer (NK) cells, despite their cytotoxic potential, often adopt a dysfunctional state. Tang et al. identified a subset of RGS1^+^NK cells enriched across cancers, characterized by impaired antitumor function and resistance to immunotherapy, potentially regulated by LAMP3^+^dendritic cells (62). Expanding on the mechanism of TuNK-mediated immunosuppression, a recent study demonstrated that TuNK cells can actively drive tumor immune tolerance (63). Specifically, TuNK cells were found to promote the accumulation and suppressive function of myeloid-derived suppressor cells (MDSCs) through the IL-6/STAT3 signaling axis, thereby facilitating tumor progression (63). Similarly, B cells can exert either immunosuppressive or anti-tumor functions depending on the tumor microenvironment, with regulatory B-cell subsets contributing to immune suppression in certain contexts. Ma et al. revealed that extrafollicular ITGAX^+^B cells are associated with poor prognosis and immunotherapy resistance by promoting a T cell-suppressive program through glutamine-derived metabolic–epigenetic crosstalk (64). In early-stage lung adenocarcinoma, IgA^+^plasma cells were found to co-localize with Tregs, suggesting a collaborative role in dampening antitumor immunity (65). CAFs also participate in immune evasion; Krishnamurty et al. demonstrated that LRRC15^+^myofibroblasts, induced by TGF-β signaling, suppress CD8^+^T cell function and impair responses to PD-L1 blockade (66). Additionally, Wang et al. identified CD300ld on neutrophils as a key mediator of tumor-induced immunosuppression by enhancing PMN-MDSC recruitment and activity via the STAT3-S100A8/A9 axis, ultimately restricting T cell activation (67).

Beyond neutrophils, Myeloid-derived suppressor cells (MDSCs) represent another formidable barrier to effective T cell immunotherapy within the solid tumor microenvironment. These pathologically activated immature myeloid cells accumulate during tumor progression and exert potent immunosuppressive effects that directly impede antitumor immunity (68). MDSCs inhibit T cell proliferation and cytotoxic function through multiple mechanisms, such as the depletion of essential amino acids (e.g., L-arginine via Arginase 1 and iNOS expression) and the production of reactive oxygen and nitrogen species (ROS and RNS) that disrupt T cell receptor signaling (68). Furthermore, MDSCs can facilitate the recruitment of regulatory T cells (Tregs) and promote tumor angiogenesis (69), collectively creating a hostile environment that limits the infiltration and efficacy of therapeutic T cells (70). Notably, this suppressive effect of MDSCs can override the enhanced function of engineered CAR-T cells, as they target the same signaling pathways modified by CAR engineering— a key mechanism underlying CAR-T clinical failure in solid tumors that is often underappreciated in preclinical development.

MDSCs are broadly classified into two major subsets based on their phenotypic and morphological features: granulocytic or polymorphonuclear MDSCs (G-MDSCs or PMN-MDSCs) and monocytic MDSCs (M-MDSCs) (68, 71). While both subsets contribute to T cell paralysis, they utilize different dominant suppressive mechanisms.

G-MDSCs, which are morphologically similar to neutrophils, primarily suppress T cells through the production of reactive oxygen species (ROS) mediated by increased NADPH oxidase (NOX2) activity. The accumulation of ROS creates an oxidative environment that induces the loss of the T cell receptor (TCR) -chain, thereby impairing T cell signaling and proliferation (68). In contrast, M-MDSCs, resembling monocytes, exert their suppressive effects predominantly through the upregulation of inducible nitric oxide synthase (iNOS) and Arginase 1 (ARG1) (68). This leads to the generation of nitric oxide (NO) and downstream reactive nitrogen species (RNS).

Critically, the interplay between ROS (from G-MDSCs) and NO (from M-MDSCs) can generate peroxynitrite (PNT), a highly reactive nitrogen species. PNT causes the nitration of tyrosine residues in the TCR-CD8 complex, interfering with the conformational flexibility required for antigen recognition and causing T cells to become unresponsive to antigen-specific stimulation (72). Collectively, these oxidative and nitrosative stress pathways orchestrated by M-MDSCs and G-MDSCs form a rigid metabolic barrier that limits the efficacy of adoptive T cell therapies.

**Figure 2:**
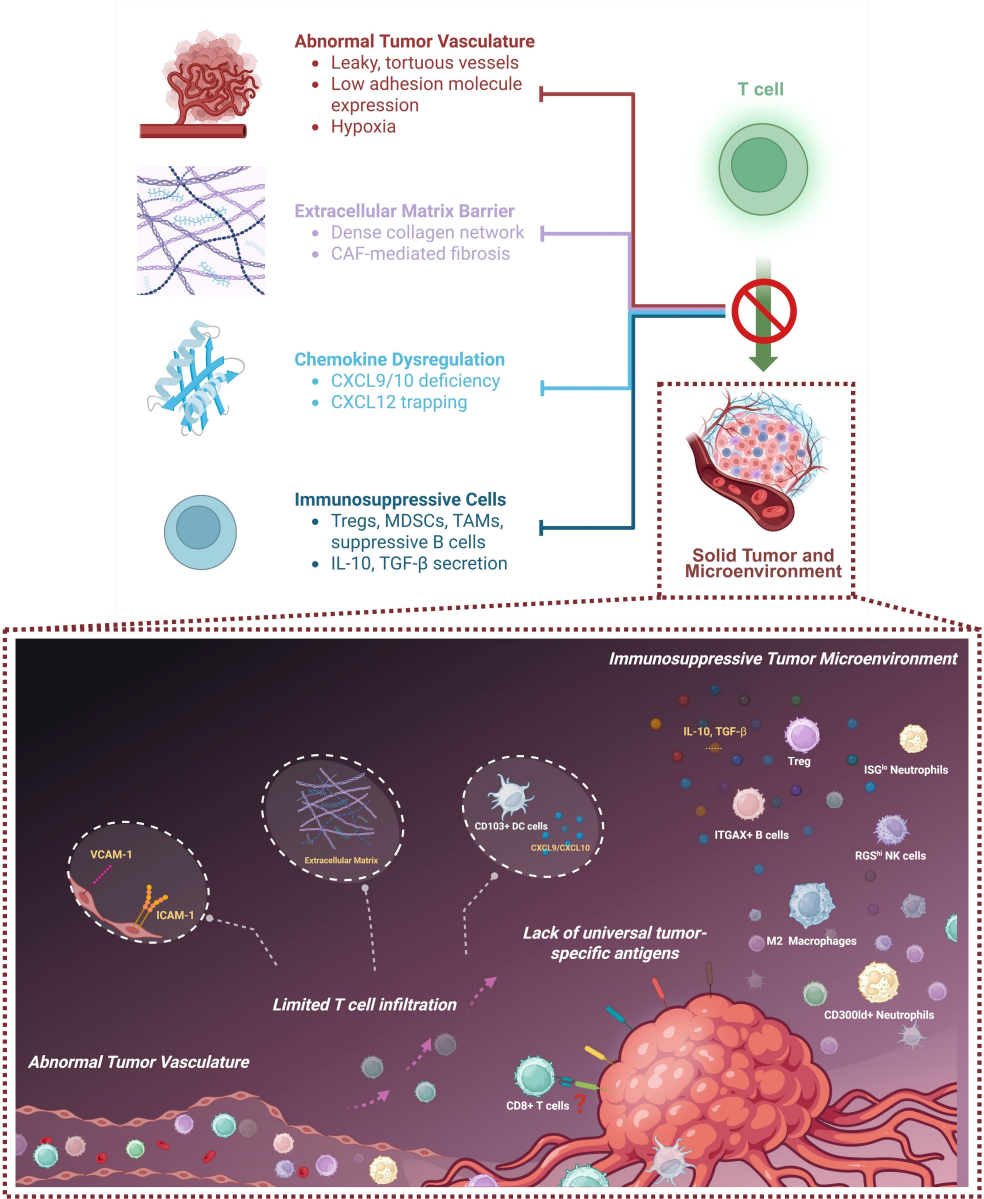
Schematic illustration of barriers to T cell infiltration in the tumor microenvironment (TME). The figure depicts factors limiting T cell infiltration into the TME, including abnormal tumor vasculature, extracellular matrix barriers, chemokine dysregulation, and the presence of immunosuppressive cells including Tregs, MDSCs, TAMs, and suppressive B cells. The inset provides a detailed view of the immunosuppressive TME and illustrates how these features collectively limit T cell infiltration and compromise T cell–based therapeutic efficacy. This figure was created in BioRender.

As summarized in **Figure 2**, these vascular, stromal, and immunologic mechanisms cooperate to exclude or inactivate effector T cells, forming a multi-layered defense that protects tumors from immune attack. Overcoming these barriers requires rationally designed combination therapies that normalize blood vessels, modulate stromal architecture, reinvigorate T cell metabolism, and block immunosuppressive circuits. Recent preclinical and clinical data suggest that targeting these pathways in concert may convert “cold” tumors into “hot” ones and improve responses to T cell–based therapies across diverse cancer types. Specifically, strategies to facilitate this conversion focus on three key aspects: priming the immune system, normalizing the tumor stroma, and removing immunosuppressive brakes. Approaches such as radiotherapy, photothermal therapy (PTT), and oncolytic viruses can induce immunogenic cell death (ICD) and release tumor antigens, thereby recruiting innate immune cells. Concurrently, vascular normalization agents can repair chaotic tumor vessels to enhance T cell trafficking, while STING agonists trigger type I interferon production to reshape the inflammatory landscape. Integrating these strategies with adoptive cell transfer holds the promise of overcoming the immune-excluded phenotype.

### 2.3 Single-cell dissection of T cell dysfunction and heterogeneity in solid tumors

T cell–based immunotherapies in solid tumors are frequently limited by profound T cell dysfunction and functional heterogeneity within the tumor microenvironment (TME). These dysfunctions encompass multiple non-mutually exclusive states, including exhaustion, senescence, and acquisition of suppressive or terminal effector phenotypes, which collectively impair T cell infiltration, persistence, and effector function. Understanding how these states arise and how they associate with therapeutic success or failure is therefore essential for elucidating the major biological obstacles to effective T cell–based therapies. For CAR-T therapy, the failure to account for TME-induced T cell dysfunction represents a critical design flaw that even exhaustion-resistant CAR-T cells engineered to downregulate PD-1 can succumb to redundant suppressive signals in solid tumor TME, leading to clinical failure.

Single-cell RNA sequencing (scRNA-seq), often combined with TCR clonotype tracing, has revolutionized our understanding of tumor-infiltrating T cell heterogeneity, developmental trajectories, and functional states. This high-resolution approach enables systematic dissection of dysfunctional T cell states within tumors and provides mechanistic insights into why T cell–based therapies succeed in some contexts but fail in others.

Across multiple cancer types, including hepatocellular carcinoma (73), non-small cell lung cancer (74), and colorectal cancer (75), scRNA-seq studies have revealed a spectrum of tumor-reactive CD8^+^T cell subsets, notably precursor exhausted T cells (Tpex), tissue-resident memory T cells (Trm), and CXCL13^+^CD8^+^T cells. Specifically, Duhen et al. identified that the co-expression of CD39 and CD103 marks a distinct population of tumor-infiltrating CD8+ T cells (CD8+ TILs) that are highly enriched for tumor reactivity in both primary and metastatic solid tumors (76). Furthermore, other phenotypes correlated with favorable prognosis and better therapeutic responsiveness have been characterized, including TCF1+ stem-like T cells (77, 78), which are essential for sustaining immune responses, and CD69+CD103+ resident memory phenotypes associated with enhanced local cytotoxicity (79, 80). These populations are often clonally expanded and associated with favorable clinical outcomes and improved responses to immune checkpoint blockade (ICB) (75, 81–84). In contrast, enrichment of terminally exhausted CD8^+^T cells or immunosuppressive subtypes such as TGF-β1^+^CD4^+^T cell, CD8^+^Temra, IL1R2^+^Tregs and KIR^+^CD8^+^T cells has been linked to poor prognosis and resistance to therapy (74, 84, 85). Preclinical models often use immunodeficient mice or syngeneic models with mild immunosuppression, failing to recapitulate the full extent of human TME-induced T cell exhaustion, which leads to overoptimistic predictions of CAR-T efficacy (19). Together, these findings highlight how distinct dysfunctional or transitional T cell states shape therapeutic responsiveness in solid tumors.

In the context of CAR-T therapy, single-cell profiling has enabled the identification of highly proliferative, memory-like T cell subsets that are predictive of therapeutic efficacy (86). For instance, TIGIT^+^CD62L^low^CD27^−^ T cells were found to give rise to the most potent CD19-specific CAR-T cells in pediatric B-ALL (87), while CD8-fit T cells with robust metabolic and migratory profiles were strongly associated with clinical responses in large B cell lymphoma (88). These findings support the prioritization of functionally competent T cell subsets during CAR-T manufacturing. Furthermore, IL-10–secreting CAR-T cells demonstrated superior metabolic resilience and durable antitumor activity in solid tumor models, offering a promising avenue to overcome exhaustion (89).

In addition to tumor-infiltrating lymphocytes, peripheral blood T cell phenotypes have emerged as complementary indicators of therapeutic competence. Patients with a higher proportion of naïve-like CD8^+^T cells in circulation exhibited better clinical outcomes in CLDN18.2-targeted CAR-T trials (90). Combined with tumor microenvironmental features such as antigen density and MYC-mediated immunosuppression, single-cell data are shaping a multi-parametric framework for responder stratification and therapy customization. In summary, single-cell technologies provide mechanistic underpinnings for therapeutic success and failure, informing the refinement of TCR-T and CAR-T therapies. By integrating transcriptional, clonal, and phenotypic data, this approach is accelerating the development of more effective, durable, and personalized T cell-based immunotherapies.

Summarily, the efficacy of T cell–based immunotherapies in solid tumors is hindered by a series of interconnected challenges, including the lack of universal tumor-specific antigens, physical and immunologic barriers that restrict T cell infiltration, and functional exhaustion of T cells within the TME. For CAR-T therapy, these challenges act synergistically to override engineering advances, and the core cause lies in the misalignment between CAR-T design assumptions (e.g., single-antigen targeting, neglect of trafficking, underestimation of TME suppression) and the biological reality of solid tumors— a mechanistic disconnect that directly drives clinical failures. From a mechanistic perspective, the inset of **Figure 2** illustrates the multiple factors that limit T cell infiltration, activation, and therapeutic efficacy within the solid tumor microenvironment. Tumor vasculature is often abnormal and poorly organized, with downregulation of key adhesion molecules such as VCAM-1 and ICAM-1, restricting T cell trafficking. The extracellular matrix forms dense physical barriers, while insufficient recruitment of CXCL9/CXCL10-producing CD103^+^dendritic cells further impair T cell homing. Tumor antigen heterogeneity complicates the identification of universal neoantigens, reducing the applicability of broadly targeted T cell therapies. In parallel, the tumor microenvironment harbors multiple immunosuppressive cell types, including regulatory T cells (Tregs) (91), ITGAX^+^B cells (64), M2 macrophages (92), CD300ld^+^neutrophils (67), ISG^lo+^neutrophils (93), and RGS^hi^ NK cells (62), which release inhibitory cytokines such as IL-10 and TGF-β, collectively suppressing T cell function, proliferation, and persistence. These interconnected factors create a hostile milieu that severely limits the clinical efficacy of adoptive T cell–based therapies in solid tumors, necessitating innovative strategies to enhance therapeutic responses.

## 3 Strategies to overcome the barriers to T cell-based immunotherapy in solid tumors

**Figure 3:**
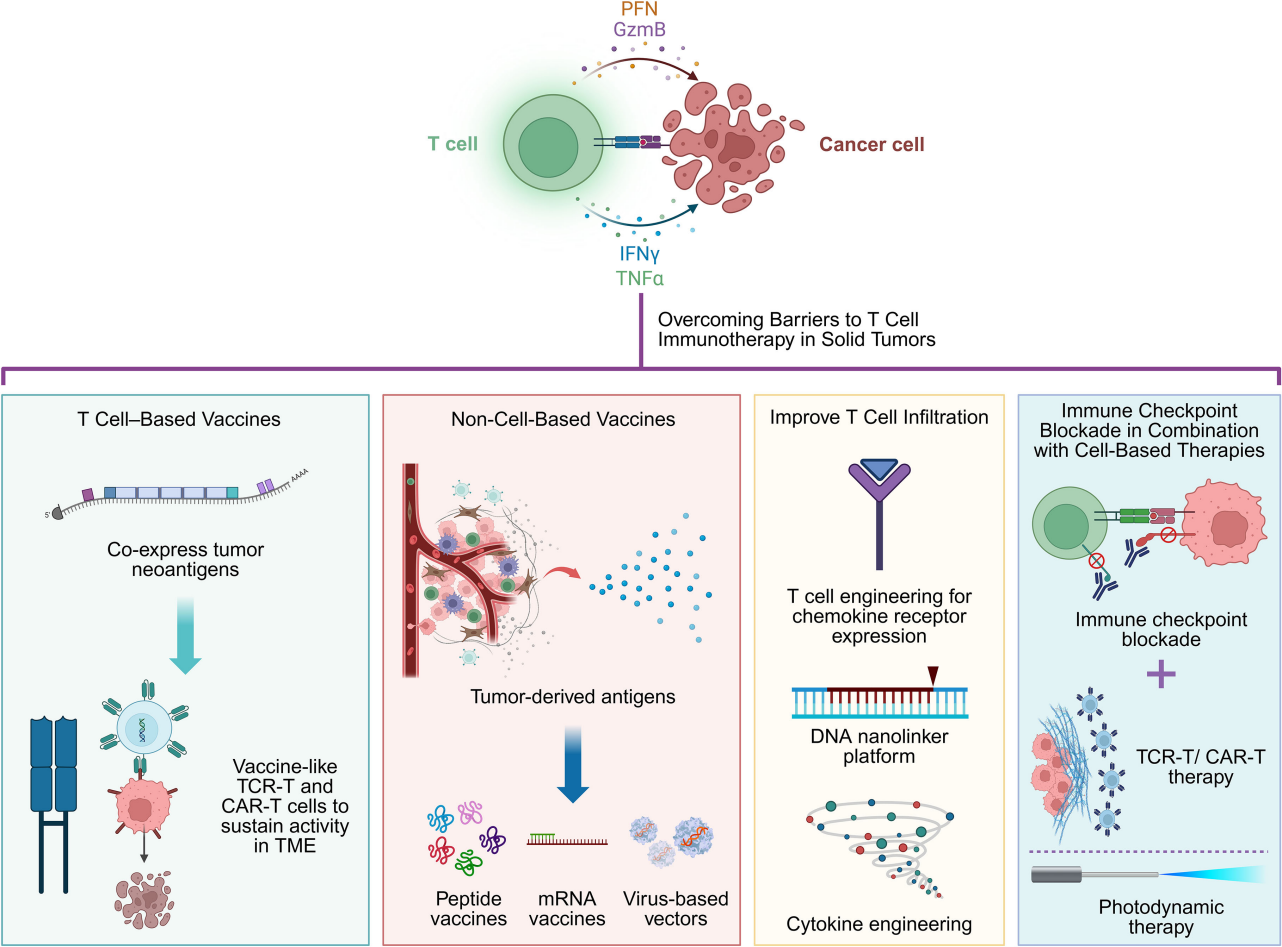
Illustration of representative strategies to overcome barriers to T cell–based immunotherapy in solid tumors. The upper panel depicts cytotoxic T cell–mediated tumor cell killing through perforin (PFN), granzyme B (GzmB), and inflammatory cytokines. The lower columns highlight the strategies to overcome barriers to T cell-based immunotherapy in solid tumors, including T cell–based vaccines that co-express tumor neoantigens to sustain TCR-T and CAR-T cell activity within the tumor microenvironment; non–cell-based vaccines derived from tumor antigens, including peptide, mRNA, and virus-based platforms; T cell infiltration improvement through chemokine receptor engineering, cytokine engineering, and DNA nanolinker–based delivery systems; and combination strategies integrating immune checkpoint blockade with cell-based therapies such as TCR-T and CAR-T cells, as well as photodynamic therapy. This figure was created in BioRender.

Despite significant obstacles to T cell-based immunotherapy in solid tumors, various innovative strategies have emerged to enhance therapeutic performance, as summarized in **Figure 3**. These approaches aim to improve antigen recognition, overcome immune suppression, enhance T cell trafficking, and ensure safety and persistence. Below, we elaborate these promising directions currently under preclinical and clinical investigation.

### 3.1 T cell–based vaccines to boost endogenous immunity

T cell-based vaccines (Tvax) represent an emerging class of cancer immunotherapy platforms that leverage the patient’s own T cells not only as effectors but also as antigen-delivering agents. In contrast to conventional vaccine approaches that rely on dendritic cells or peptide formulations, Tvax utilizes autologous T cells genetically engineered to express tumor-associated antigens and immunostimulatory molecules. These modified T cells traffic to lymphoid tissues, where they promote antigen cross-presentation and amplify endogenous T cell responses, effectively functioning as cellular adjuvants.

A landmark study by Veatch et al. established the principle of Tvax by engineering patient-derived T cells to co-express tumor neoantigens alongside immunomodulatory factors such as CD80, IL-12, and GM-CSF. This approach resulted in robust CD4^+^and CD8^+^T cell activation through dendritic cell engagement and antigen cross-presentation. Notably, the combined expression of IL-12 and GM-CSF led to the most potent T cell priming effects, highlighting the potential of Tvax to bridge innate and adaptive immunity (94, 95). Building upon this concept, recent efforts have focused on equipping therapeutic T cells, including TCR-T and CAR-T cells with additional “vaccine-like” functionalities to sustain their activity in the tumor microenvironment. For example, synthetic cytokine receptors such as engineered IL-21R allow T cells to maintain long-term STAT3 activation, thereby enhancing memory formation and resistance to exhaustion (96). Similarly, co-expression of membrane-tethered IL-15 and IL-21 in engineered T cells has demonstrated broad efficacy in epithelial tumor and neuroblastoma pre-clinical models, establishing a modular strategy for T cell armoring (97). Furthermore, LAG-3Ig, TCR cognate peptides, and tumor neoantigen-engineered T cell vaccines exerted a synergistic boosting effect on TCR-T cells, thereby enhancing their antitumor activity against heterogeneous MC38 colorectal carcinoma in murine models (98).

Chemokine and cytokine engineering strategies have also proven effective in improving the tumor accessibility and persistence of therapeutic T cells. For instance, T cells modified to express the chemokine receptors CCR2b or CXCR2 respond to tumor-derived CCL2 or IL-8, significantly enhancing tumor homing and local cytotoxicity in malignant pleural mesothelioma and melanoma preclinical model (99, 100). Moreover, CAR-T cells co-expressing IL-7 and CCL19 have been shown to reconstitute lymphoid-like niches within the tumor microenvironment, thereby promoting dendritic cell and T cell infiltration, improving CAR-T cell survival, and establishing durable immune memory in B16F10 melanoma cells and Lewis Lung Carcinoma cells mouse models (101, 102).

DCs play a vital role in processing antigens and priming T cells for an effective anti-tumor immunity, leading to tumor regression. Notably, accumulating evidence has demonstrated that CAR-T therapy can actively promote DC recruitment to the tumor microenvironment. Specifically, Ma et al. reported in a landmark study published in Cell that vaccine-boosted CAR-T cells significantly enhanced DC recruitment to tumors, increased tumor antigen uptake by DCs, and further elicited the priming of endogenous anti-tumor T cells (103). This process is critically dependent on IFN-γ secreted by CAR-T cells, and genetic amplification of IFN-γ expression in CAR-T cells can further strengthen this effect, thereby enhancing the control of heterogeneous tumors. Depletion experiments in murine tumor models confirmed that DCs are indispensable for the enhanced anti-tumor efficacy induced by vaccine-boosted CAR-T cells, highlighting the crucial crosstalk between CAR-T therapy and DC recruitment in amplifying anti-tumor immunity.

In addition to enhancing infiltration, strategies have also been developed to counteract the suppressive signals within the tumor milieu. A notable example is the use of inverted cytokine receptors (ICRs), which convert inhibitory signals such as TGF-β into stimulatory signals that promote T cell survival and expansion. One such ICR, TB15, incorporates the extracellular domain of TGF-βRII fused with the intracellular signaling domain of IL-15Rα, allowing engineered CAR-T cells to thrive in TGF-β–rich environments by activating pro-survival pathways (104). These innovative signaling rewiring strategies represent a major advance in enabling engineered T cells to overcome immune suppression and persist in mouse colon cancer.

Furthermore, engineered T cells secreting Flt3L have been shown to expand intratumoral dendritic cells and initiate *de novo* immune responses, acting as self-adjuvanting cellular vaccines (105). Importantly, clinical observations have also confirmed that mutation-specific CD4^+^T cells can mediate tumor regression and broaden epitope recognition, further supporting the concept of leveraging engineered T cells as therapeutic immunization agents (106). Altogether, Tvax offer a powerful paradigm for immunotherapy, uniquely positioned to combine targeted tumor killing with systemic immune education. By integrating vaccine functions directly into engineered T cells, Tvax platforms provide a promising solution to overcome immunosuppression, enhance immune memory, and reduce antigen escape in solid tumors. This concept stands in contrast to conventional, non–T cell–based vaccines and represents a new frontier in adoptive cellular immunotherapy.

### 3.2 Non-cell-based vaccines for cancer immunotherapy

In parallel with T cell-based therapies, non–cell-based cancer vaccines have emerged as a versatile and increasingly validated strategy to elicit tumor-specific immune responses. By delivering tumor-derived antigens directly *in vivo*, these platforms aim to stimulate endogenous T cell immunity with greater flexibility and scalability than cellular therapies. Among these, peptide vaccines, mRNA vaccines, and virus-based vectors have shown notable clinical progress in recent years.

Peptide-based neoantigen vaccines constitute one of the earliest forms of personalized cancer vaccines. These vaccines are typically composed of multiple long peptides derived from patient-specific tumor mutations, identified through next-generation sequencing and HLA binding prediction algorithms (107). Notably, NEO-PV-01, developed by Neon Therapeutics, demonstrated encouraging immune activation when combined with PD-1 blockade and chemotherapy in non–small cell lung cancer, reinforcing the potential of personalized peptide vaccines in first-line combination settings (108). Building on this, the NeoVax study in melanoma patients showed that vaccination induced durable memory T cell responses and epitope spreading, even four years post-treatment, suggesting possible long-term protective immunity (109). Importantly, similar peptide-based strategies were also applied in glioblastoma, where neoantigen vaccines successfully elicited intratumoral immune responses in a typically immune-excluded tumor context (110). In the landmark NeoVax studies, DCs play a pivotal role—they capture and cross-present NeoVax’s neoantigen peptides to naive CD4^+^/CD8^+^T cells, triggering durable tumor-specific immune responses with the help of adjuvant poly-ICLC that enhances DC activation and antigen presentation capacity. Currently, a Phase Ib study (NCT04930783) is investigating the combinatorial strategy of NeoVax with CDX301 and Nivolumab/Pembrolizumab for melanoma. As a recombinant Flt3 ligand, CDX301 regulates DC proliferation and activity to strengthen antigen presentation, while Nivolumab/Pembrolizumab reinvigorates exhausted T cells. This synergy amplifies NeoVax-induced immune responses and improves therapeutic efficacy. These findings collectively highlight the feasibility, safety, and immunogenicity of long-peptide vaccines across diverse tumor types.

While peptide vaccines provide precise epitope delivery, mRNA-based vaccines offer enhanced flexibility, rapid manufacturing, and multi-epitope encoding in a single formulation. One of the earliest clinical demonstrations came from Sahin and colleagues, who designed personalized RNA mutanome vaccines targeting multiple patient-specific mutations in melanoma. This approach successfully induced broad poly-specific T cell responses, with two patients showing objective tumor regression and one achieving complete remission when combined with anti–PD-1 therapy. Tumor biopsy analysis further confirmed that vaccine-induced T cells infiltrated the tumor and recognized autologous targets, thus validating the concept of RNA-based neoantigen vaccination in humans (111). Building on this foundation, early-phase trials of personalized mRNA vaccines encoding patient-specific neoantigens or recurrent driver mutations (e.g., KRAS G12D) have shown that they can induce T cell responses in gastrointestinal cancers, with favorable safety profiles (112). Going a step further, the BNT211 trial combined CLDN6-targeted CAR-T cells with an mRNA vaccine (CARVac), achieving not only high CAR-T expansion and engraftment, but also a 33% objective response rate in heavily pretreated solid tumor patients (113). In the adjuvant setting, mRNA vaccines are also gaining ground. For instance, the KEYNOTE-942 trial showed that mRNA-4157 (V940) combined with pembrolizumab significantly prolonged recurrence-free survival in resected high-risk melanoma compared to pembrolizumab alone (114). These results collectively suggest that mRNA vaccines can function both as standalone immunotherapies and as amplifiers of other modalities, such as CAR-T or immune checkpoint blockade.

In addition to peptide and mRNA vaccines, oncolytic viruses (OVs) represent a unique class of non-cell-based immunotherapy that combines direct tumor cell lysis with *in situ* antigen release and immune priming. Modern OV designs increasingly incorporate immunomodulatory payloads to boost therapeutic efficacy. For example, a recombinant HSV-1 virus encoding a cetuximab-CCL5 fusion protein (OV-Cmab-CCL5) was shown to suppress EGFR-positive glioblastoma growth by recruiting innate and adaptive immune cells into the tumor microenvironment (115). Moreover, clinical experience with agents such as T-VEC and ongoing trials with new OV candidates underscore both the therapeutic potential and the translational challenges of this platform, including immune evasion and delivery efficiency (116). Nevertheless, their compatibility with other immunotherapies makes OVs a promising component of multi-modal treatment strategies.

Taken together, these advances in peptide, mRNA, and virus-based cancer vaccines underscore the increasing relevance of non–cell-based platforms in the immunotherapy landscape. While each modality has distinct strengths, their integration with T cell therapies, checkpoint inhibitors, and tumor-targeted approaches may offer synergistic benefits. Moving forward, improvements in neoantigen prediction, delivery vectors, and combination regimens will be pivotal in optimizing their clinical utility across both early-stage and metastatic cancers.

### 3.3 Strategies to improve T cell infiltration in solid tumors

Despite the remarkable success of CAR T cell therapy in hematologic malignancies, its efficacy in solid tumors remains limited, largely due to poor T cell infiltration. Solid tumors are often characterized by physical barriers such as dense extracellular matrix (ECM), an immunosuppressive TME, and chemokine mismatch, all of which collectively hinder T cell trafficking, survival, and antitumor activity within the tumor core (117, 118).

Various strategies have been developed to overcome these challenges and redefine the TME (119). One key approach involves engineering T cells to express chemokine receptors that match tumor-secreted chemokines, thereby improving homing and accumulation within the tumor (117). For example, forced expression of CXCR2 or CCR2b on CAR-T cells enables response to CXCL8 or CCL2, respectively, significantly enhancing migration into the tumor microenvironment (120). Moreover, remodeling the extracellular matrix is another promising tactic. Overexpression of MMP-7 or SPP1 (osteopontin) in GD2-CAR-T cells has been shown to improve extravasation and interstitial migration through ECM-dense regions in neuroblastoma models, resulting in significantly enhanced tumor control without increased off-tumor toxicity (121). Co-stimulatory receptor optimization has also shown efficacy. A study revealed that OX40 co-expression enhances CAR-T infiltration by promoting heparan sulfate-mediated adhesion, leading to improved tumor localization and persistence (122). Similarly, CD93 blockade was found to upregulate vascular adhesion molecules, facilitate effector T cell infiltration, and enhance CAR-T efficacy in melanoma models (123).

In addition to receptor-level modifications, some researchers have focused on intercellular cooperation. A novel DNA nanolinker platform connects T cells with tumor-homing monocytes, leveraging monocyte infiltration pathways to transport T cells into tumor sites. This “monocyte hitchhiking” strategy significantly enhanced T cell accumulation and intratumoral recruitment in breast cancer and melanoma models (124). Synthetic receptor systems also offer precise control over T cell function. For example, STAR-T cells, which integrate antibody-based antigen recognition with native TCR signaling machinery, have demonstrated enhanced antigen sensitivity, resistance to exhaustion, and superior antitumor effects in multiple solid tumor models (125). Synthetic circuits such as synNotch further enable context-dependent cytokine release and spatially restricted activation, thereby indirectly supporting infiltration and persistence (126).

Furthermore, advances in cytokine engineering have contributed to overcoming TME-induced dysfunction. Strategies include integrating cytokine genes (e.g., IL-7, IL-15, IL-18) or synthetic cytokine receptors to enhance T cell persistence and resistance to suppression (120). Combination therapies, such as CAR-T plus checkpoint inhibitors or anti-fibrotic agents, are also being explored to further disrupt physical and immunological barriers (127). Together, these intrinsic engineering approaches aim to enhance T cell access to tumor cores while maintaining functional durability.

Meanwhile, approaches originating from other scientific disciplines are being actively investigated to address existing barriers and enhance the efficacy of T cell-based immunotherapies. For example, photodynamic therapy (PDT) and photothermal therapy (PTT) have been explored to modulate the TME, especially the immunosuppressive cell populations (128). For instance, a design of PDT/PTT nanoplatform loads catalase (CAT) and an anti-GITR antibody (DTA-1) onto PDA–ICG photothermal–photosensitizer nanoparticles to target the constitutive expression of GITR on Tregs (129), while another design further enhances PDT/PTT efficacy to elicit potent tumor-specific T cell responses and overcome immunological resistance through synergistic activation of the cGAS–STING pathway and immunogenic cell death (ICD) (130). ICD also helps reverse immunosuppression and enhance antitumor immune responses when combined with gemcitabine (GEM)–mediated depletion of myeloid-derived suppressor cells (MDSCs) (131). In a further example, an NIR-II–responsive degradable pseudo-conjugated polymer (PSP)–based PDT system co-delivers regorafenib, which is released under 808 nm laser irradiation to normalize tumor vasculature, alleviate hypoxia, and enhance reactive oxygen species (ROS) generation for antitumor activity (132), which can be amplified by integration with polymer-encapsulated carbonized hemin nanoparticles (P-CHNPs) (133).

Besides directly intervening in the microenvironment of solid tumors, PDT and PTT also have the potential to enhance T cell infiltration by softening the tumor ECM (134, 135) and improving blood flow and vascular permeability (136). Notably, PDT and PTT have distinct drawbacks: PDT can exacerbate intratumoral hypoxia, whereas PTT upregulates heat shock protein (HSP) expression (137). Consequently, combining the two modalities is actively being explored, with PTT alleviating hypoxia and PDT decomposing HSPs, thereby mitigating each other’s limitations (138).

### 3.4 Immune checkpoint blockade in combination with other therapies

The integration of immune checkpoint blockade (ICB) with adoptive cell therapies such as TCR-T or CAR-T has emerged as a promising yet complex approach to enhancing antitumor immunity, particularly in solid tumors. Immune checkpoints including PD-1, CTLA-4, LAG-3, TIM-3, and TIGIT exert distinct regulatory effects across T cells, NK cells, and dendritic cells, necessitating precise selection and timing in combinatorial strategies (139). Moreover, FGL1 has recently been identified as a key LAG-3 ligand involved in immune escape and PD-1 resistance, offering a potential new axis for combination therapy (140).

Accumulating evidence indicates that ICB most effectively amplifies endogenous, vaccine-primed antitumor T cell responses, whereas its ability to reinvigorate already-dysfunctional engineered T cells remains inconsistent. Several studies have shown that ICB can synergize with cancer vaccines. Liu et al. demonstrated that combining neoantigen vaccines with PD-1 blockade expanded cytotoxic CD8^+^T cell subsets with low inhibitory receptor expression and enhanced chemokine signaling (141). Puig-Saus et al. showed that durable responses to PD-1 therapy in melanoma were associated with persistent, polyclonal neoantigen-specific CD8^+^T cells identified using single-cell and CRISPR-based profiling (142). In hepatocellular carcinoma, Yarchoan et al. found that a DNA neoantigen vaccine combined with IL-12 plasmid and pembrolizumab achieved a 30.6% objective response rate and induced robust CD4^+^and CD8^+^T cell responses in 86.4% of patients (143). Collectively, these studies support the notion that ICB primarily enhances *de novo* or vaccine-induced T cell immunity rather than reversing established dysfunction.

However, the efficacy of ICB in combination with engineered T cells remains less predictable. Stromnes et al. found that TCR-T cells targeting pancreatic tumors rapidly developed transcriptional features of exhaustion that were refractory to checkpoint blockade, suggesting that T cell dysfunction was rooted in both *in vitro* programming and suppressive tumor environments (144). Davies et al. further demonstrated that PD-1 blockade improved tumor control mainly by stimulating endogenous T cells rather than reversing the dysfunction of transferred TCR-T cells, indicating a lack of true synergy in such contexts (145).

Concurrently, ICB can also be enhanced by interdisciplinary approaches, including the aforementioned PDT method. For instance, He et al. demonstrated that PDT could contribute to an immunogenic environment in the tumour, which significantly enhanced PD-L1 checkpoint blockade therapy by generating systemic antitumor immunity (146). Such physical modulation strategies also offers possibility of increasing applicability and operability through miniature optical devices (147).

Another notable example of ICB enhancement is its combination with dietary intervention, which can modulate the gut microbiome and thereby influence therapeutic outcomes (148–151). Short-chain fatty acids (SCFAs) have been linked to ICB efficacy, though both positive (152) and negative (153) associations have been reported. Dietary fiber is another key modulator of the gut microbiome, with lower fiber intake observed to correlate with reduced ICB response (154). A phase II clinical trial (NCT04645680) is currently evaluating the impact of a controlled high-fiber dietary intervention on ICB efficacy in melanoma patients, with its findings expected to provide important translational insights into the role of dietary interventions in cancer immunotherapy (154, 155).

These results collectively emphasize that ICB can markedly enhance vaccine-induced endogenous T cell responses but may have limited efficacy in reinvigorating already-dysfunctional engineered T cells. Notably, integrating ICB with other physical modalities such as PDT has shown the potential to potentiate antitumor immunity. Future strategies should consider temporally staged or context-specific ICB administration, possibly in combination with synthetic modifications to TCR/CAR constructs and adjunctive therapies like PDT/PTT, to fully unlock the combinatorial potential in solid tumor immunotherapy.

### 3.5 Clinical progress of T cell–based therapies across solid tumor types

**Figure 4:**
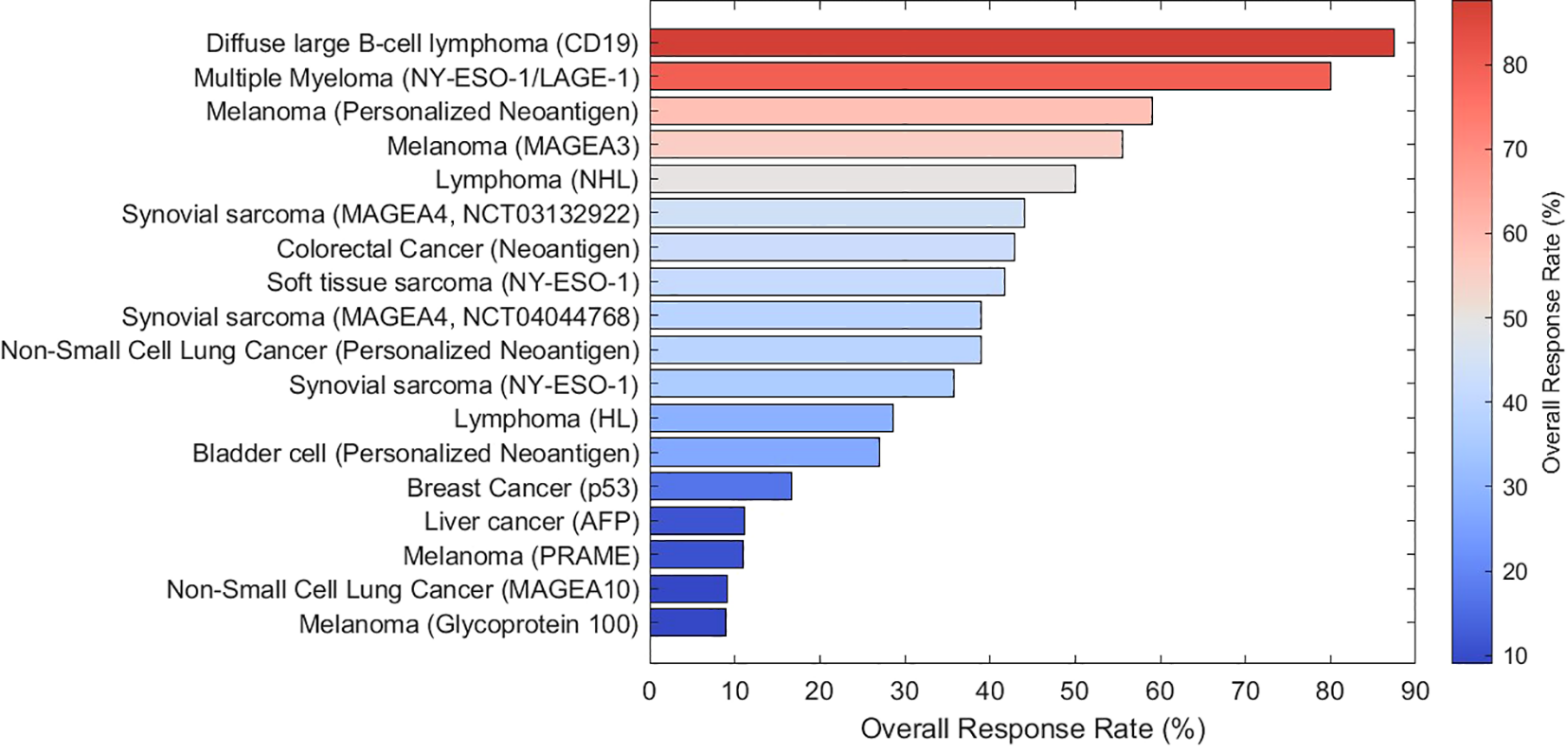
Summary of reported overall response rates (ORRs) of T cell–based immunotherapies across different cancer types and target antigens. This figure visualizes the clinical outcomes in multiple types of hematological malignancies and solid tumors. Bars indicate the ORR reported in representative studies, with color intensity reflecting response magnitude. This figure was plotted in MATLAB.

Significant progress has been made in translating T cell-based immunotherapies to solid tumors through improved target identification, cell engineering, and clinical trial design. Although response rates remain variable across cancer types, emerging clinical evidence suggests that, in specific contexts, adoptive T cell therapies can induce durable remissions. This section highlights recent developments in several major solid tumor types, as summarized in **Table 1**, with the corresponding overall response rates (ORRs) visualized in **Figure 4**.

**Table 1:** Summary of clinical and preclinical studies of T cell-based therapies across cancer types.

Cancer type	Antigen target	Clinical trial number	Phase	Efficacy	ORR	Ref
Lymphoma	PRAME, SSX2, MAGEA4, SURVIVIN, NY-ESO-1	NCT01333046	I	Durable complete remissions occurred in 6 of 15 chemorefractory lymphoma patients, including 2 with Hodgkin and 4 with non-Hodgkin lymphoma.	NHL:50% HL:28.6%	(156)
Diffuse large B-cell lymphoma	CD19	NCT04014894	I	OS, PFS and DOR at 3 years were 75.0%, 62.5%, and 71.4%, respectively.	87.5%	(157)
Multiple myeloma	NY-ESO-1/LAGE-1	NCT01352286	I/II	Median PFS was 19.1 months	80%	(158)
	BTN3A1(TEG001)	NTR6541/NL6357	I	The single patient did not demonstrate a reported objective response.	–	(188–190)
Synovial sarcoma	NY-ESO-1	NCT01343043	I/II	NY-ESO-1 SPEAR T cells induced responses in 15 of 42 synovial sarcoma patients, with efficacy associated with T cell persistence, high NY-ESO-1 expression, and fludarabine-based conditioning.	35.7%	(160, 161)
	NY-ESO-1 TCR/IL-15 NK	NCT06083883	I/II	Undisclosed	–	(191)
	MAGEA4	NCT04044768	II	The median DOR was 11.6 months.	39%	(164)
		NCT03132922	I	The DOR was 25.6 weeks.	44%	(163)
Soft tissue sarcoma	NY-ESO-1	NCT04318964	I	The median PFS was 7.2 months, and the median DOR was 13.1 months.	41.7%	(192)
Melanoma	Glycoprotein 100	NCT03070392	III	The median OS was 21.7 months, and the median DOR was 9.9 months.	9%	(168)
	MAGEA3	NCT01273181	I/II	This study reveals low-level MAGE-A expression in normal brain, underscoring the neurotoxicity risk of TCR therapy	55.6%	(171)
	PRAME	NCT04262466	I/II	The median PFS was 4.2 months.	11%	(169)
	Personalized Neoantigen	NCT02897765	Ib	The median PFS was 23.5 months and 1-year OS rate was 96%.	59%	(170)
Colorectal cancer	Neoantigen	NCT03412877	II	Neoantigen-specific TCR-T cell therapy induced objective responses in 3 of 7 metastatic colorectal cancer patients, with regressions lasting up to 7 months.	42.9%	(172)
	KRAS G12V	NCT06105021	I	Undisclosed	–	(175)
	KRAS G12D	–	Preclinical	Adoptive transfer of CD8^+^T cells targeting mutant KRAS G12D induced objective regression of all lung metastases in a metastatic colorectal cancer patient, though relapse occurred due to tumor immune evasion via HLA-C*08:02 loss.	–	(173)
Liver cancer	AFP	NCT01312792	I	One complete response, and a disease control rate of 64% with stable disease for ≥16 weeks in two patients,	11.1%	(176, 177)
	GPC3	NCT05926726	I	Undisclosed	–	(179)
	AFP158	–	Preclinical	AFP158-specific TCR-T cells eradicated HCC xenografts in mice with minimal off-target activation, supporting their promising efficacy and safety.	–	(178)
Breast cancer	p53	NCT00068003, NCT01174121, NCT03412877	II	TCR-based adoptive cell therapy targeting shared TP53 mutations showed limited efficacy with TILs but achieved a 55% tumor reduction lasting 6 months in one patient using a transduced TCR, supporting its potential for broader application.	16.7%	(180)
	PLAC1		Preclinical	A novel PLAC1-specific, HLA-A2–restricted TCR showed antigen-specific cytotoxicity and delayed tumor growth in breast cancer models.	–	(181)
Ovarian cancer	Mesothelin	NCT03907852,	I/II	Mesothelin-targeted TRuC-T cells showed superior tumor rejection kinetics and metabolic fitness over CAR-T cells in solid tumor models, indicating promising efficacy for treating mesothelin-expressing cancers.	–	(182)
	MAGEA4+CD8	NCT04044859	I	Undisclosed	–	(184)
	NY-ESO-1	–	Preclinical	The 19305DP-derived TCR enabled both CD4^+^and CD8^+^T cells to effectively eliminate NY-ESO-1^+^tumors *in vivo* without detectable off-target reactivity.	–	(183)
Clear cell renal cell carcinoma	HERV-E	NCT03354390	I	Undisclosed	–	(185)
Bladder cell	Personalized Neoantigen	NCT02897765	Ib	The median PFS was 5.8 months, and the median OS was 20.7 months, with a 1-year OS rate of 67%.	27%	(170)
Non-small cell lung cancer	MAGEA10	NCT02592577	I	Among 11 patients, one achieved aPR, four had SD, and the therapy showed dose-dependent persistence in blood and tumor tissue.	9.1%	(186)
	Personalized Neoantigen	NCT02897765	Ib	The median PFS was 8.5 months with 1-year OS rate was 83%	39%	(170)
Solid tumor	p53, KRAS, EGFR	NCT05194735	I/II	Undisclosed	–	(187)

This table provides an overview of representative clinical and preclinical studies involving T cell-based immunotherapies across a broad spectrum of malignancies. Each entry includes the cancer type, antigen target, clinical trial identifier (NCT number), trial phase, descriptive efficacy outcomes (e.g., complete response, progression-free survival), and overall response rate (ORR) when available. The table integrates both ongoing and completed trials, and incorporates select preclinical data to illustrate therapeutic potential in early development stages. Trial phases range from first-in-human studies to late-stage Phase II/III trials. NHL, non-Hodgkin lymphoma; HL, Hodgkin lymphoma; OS, overall survival; PFS, progression-free survival; DOR, duration of response; CR, complete response; PR, partial response; SD, stable disease; DCR, disease control rate.

#### 3.5.1 Hematologic malignancies

T cell therapies have shown considerable promise across various hematologic malignancies, including lymphomas, acute myeloid leukemia (AML), and multiple myeloma (MM). In lymphoid malignancies, a phase I study using autologous non-engineered T cells targeting five tumor-associated antigens (PRAME, SSX2, MAGE-A4, SURVIVIN, and NY-ESO-1) demonstrated an ORR of 50% in non-Hodgkin lymphoma (NHL) and 28.6% in Hodgkin lymphoma (HL), with some complete responses lasting over three years (156). In CD19-positive B cell malignancies, the γδ TCR-based therapy ET019003 achieved an ORR of 87.5% and a median overall survival exceeding three years, underscoring the potential of unconventional TCR platforms (157). In contrast, TCR-T therapies in multiple myeloma have yielded encouraging results. NY-ESO-1/LAGE-1–specific T cells infused post–stem cell transplant led to an 80% ORR and a median progression-free survival of 19.1 months, supported by evidence of *in vivo* expansion, bone marrow homing, and sustained cytotoxicity (158).

#### 3.5.2 Synovial and soft tissue sarcoma

Synovial sarcoma represents one of the most responsive solid tumors to TCR-T cell therapy, largely due to its high and homogeneous expression of cancer-testis antigens such as NY-ESO-1 and MAGE-A4 (159). Early studies targeting NY-ESO-1 demonstrated durable clinical activity. In a clinical trial, autologous TCR-T cells targeting NY-ESO-1 SPEART achieved a 37.5% objective response rate (ORR) in patients with advanced synovial sarcoma, with functional persistence of transferred T cells lasting over 6 months (160, 161). Subsequent translational analyses revealed that the antitumor effect was associated with both systemic and local immune activation, including infiltration of CD8^+^T cells and inflammatory myeloid cells, despite the immunologically “cold” nature of the tumor.

More recently, MAGE-A4 has emerged as another promising target (162). The autologous TCR-T product afamitresgene autoleucel (afami-cel) targets MAGE-A4 in HLA-A*02:01–positive patients. In a phase I trial involving 38 patients with advanced solid tumors, afami-cel showed an ORR of 44% in synovial sarcoma, with a median duration of response of 25.6 weeks. Treatment-related adverse events primarily consisted of grade ≥3 cytopenias, occurring in 55% of patients, and cytokine release syndrome (CRS) in 13% of patients, the majority of which were grade 1–2 in severity (163). The subsequent SPEARHEAD-1 phase II study further confirmed the clinical benefit in this population. Among 52 evaluable patients, the confirmed ORR was 39%, and the median duration of response reached 11.6 months, with responses observed even in patients with bulky or metastatic disease (164). These findings support the continued development and potential regulatory advancement of afami-cel for synovial sarcoma, which remains a high-need, antigen-defined solid tumor. And Afami-cel has since been approved for the treatment of advanced synovial sarcoma, marking a significant milestone for TCR-T cell therapies (165).

#### 3.5.3 Melanoma

Tebentafusp, a novel bispecific TCR therapeutic targeting glycoprotein 100 (gp100) and CD3, is the first TCR therapy approved for metastatic uveal melanoma (166). Gp100 is an antigen molecule that first appears during early embryonic development. It is considered a melanoma-associated antigen, with its expression significantly upregulated during melanoma progression (167). Tebentafusp achieved a median OS of 21.7 months despite a modest 9% ORR (168). In addition, Brenetafusp is an engineered ImmTAC molecule designed to redirect T cells toward PRAME-expressing tumors in HLA-A*02:01–positive patients. In early-phase clinical trials, it demonstrated an objective response rate (ORR) of 11% and a median progression-free survival (mPFS) of 4.2 months in melanoma, indicating its potential as a novel targeted immunotherapy (169). Personalized neoantigen vaccines, such as NEO-PV-01 in combination with PD-1 blockade, have shown encouraging efficacy in patients with melanoma, achieving an objective response rate (ORR) of 59%. The median progression-free survival (PFS) was 23.5 months, and the 1-year overall survival (OS) rate reached 96% (170). Nevertheless, safety remains a critical concern; early clinical investigations of TCR-T therapies targeting MAGE-A3 revealed severe neurotoxicity attributed to off-tumor antigen expression, emphasizing the importance of rigorous antigen specificity validation prior to clinical application (171).

#### 3.5.4 Gastrointestinal cancers

Adoptive T cell therapies targeting mutated KRAS or neoantigens are gaining momentum in colorectal cancer (CRC). A landmark phase II trial of personalized TCR-T cells targeting patient-specific neoantigens achieved a 42.9% ORR with responses lasting 4–7 months (172). More recently, a phase I trial conducted by Tran et al. demonstrated the clinical efficacy of tumor-infiltrating lymphocytes (TILs) targeting KRAS G12D, leading to complete regression of all lung metastases in a patient with metastatic colorectal cancer. However, despite the initial response, disease relapse occurred due to tumor immune evasion mediated by the loss of HLA-C*08:02 expression (173). Notably, recent advances in precision TCR engineering have addressed key limitations of traditional KRAS-targeted T cell therapies. Zheng et al. reported the development of high-affinity, precision-engineered TCRs specifically recognizing KRAS G12D (174). This work employed rational TCR discovery strategies guided by structural modeling of the KRAS G12D-derived peptide-HLA complex, followed by affinity maturation engineering to enhance TCR binding avidity. Importantly, these engineered TCRs exhibit well-defined HLA restriction to HLA-A*11:01, providing critical guidance for patient stratification in clinical translation, and have undergone comprehensive safety validation, including *in vitro* assessments of off-target reactivity to ensure minimal cross-reactivity with non-tumor tissues. Moreover, multiple KRAS-targeted TCR-T candidates are in early-phase clinical development (175).

Additionally, in liver cancer, clinical studies with AFP-specific products like ADP-A2AFP have begun reporting modest 11.1% ORR. Among nine patients, one patient achieved a complete response, and 64% of patients experienced disease control, including stable disease lasting at least 16 weeks in two cases (176, 177). And AFP-targeting TCR-T therapies have shown encouraging preclinical safety data. *In vitro* analyses confirmed low cross-reactivity with non-tumor tissues (178). Other HCC-directed targets such as GPC3 and HBV antigens are currently being investigated in early-phase trials (179).

#### 3.5.5 Gynecologic and breast cancers

In breast cancer, TCR-T therapies targeting mutant p53 have entered clinical trials. One patient experienced a 55% tumor reduction lasting 6 months, although 16.7% ORR remain moderate (180). Additionally, PLAC1-specific TCR-T cells have shown potent cytotoxicity *in vitro* and significant tumor suppression in xenograft models, supporting PLAC1 as a viable target (181). These data suggest that target selection and cell fitness are critical in this setting.

In parallel, TCR-T therapies targeting mesothelin or NY-ESO-1 are in preclinical and early clinical stages for ovarian cancer. TRuC-T cells (e.g., TC-210) demonstrated superior function and persistence over conventional CAR-T cells in preclinical models (182). Notably, dual-positive (CD4^+^CD8^+^) T cells expressing CD8-independent, high-affinity NY-ESO-1–specific TCRs have shown broad cytotoxicity *in vitro* and *in vivo*, offering an unconventional TCR source for future engineering (183). Furthermore, ADP-A2M4CD8 is a TCR-T cell therapy targeting melanoma-associated antigen A4 (MAGE-A4), engineered with a CD8α co-receptor, for the treatment of patients with HLA-A*02–positive unresectable or metastatic solid tumors. In the ongoing phase 1 SURPASS trial, ADP-A2M4CD8 monotherapy has demonstrated an acceptable benefit-to-risk profile and encouraging antitumor activity (184).

#### 3.5.6 Other solid tumors

Several early-phase clinical trials have demonstrated preliminary clinical activity of TCR-T cell therapies across a range of solid tumors. In clear cell renal cell carcinoma, a phase I trial targeting HERV-E has been initiated, although efficacy data remain undisclosed to date (185). In bladder cancer, a phase Ib study evaluating a personalized neoantigen vaccine combined with PD-1 blockade reported a median progression-free survival (PFS) of 5.8 months and a median overall survival (OS) of 20.7 months, with a 1-year OS rate of 67% and an ORR of 27% (170). For non-small cell lung cancer (NSCLC), a phase I trial of MAGEA10-targeted TCR-T cells showed an ORR of 9.1% among 11 patients, including one PR and four cases of SD, with dose-dependent persistence observed in both blood and tumor tissues (186). Additionally, in NSCLC patients treated with personalized neoantigen vaccines, the ORR reached 39%, with a median PFS of 8.5 months and a 1-year OS rate of 83% (170). In addition, a phase I/II trial is currently investigating TCR-T cell therapies targeting shared oncogenic mutations such as p53, KRAS, and EGFR across a range of solid tumors (187). While efficacy outcomes have not yet been disclosed, this trial reflects the growing interest in targeting common neoantigens to develop more broadly applicable TCR-based immunotherapies. Collectively, these findings highlight the potential of TCR-based therapies and neoantigen-targeted approaches in solid tumors and warrant further clinical investigation.

## 4 Conclusion and outlook

In this review, we have examined the key barriers limiting the efficacy of T cell-based immunotherapies in solid tumors, including tumor antigen heterogeneity, impaired T cell infiltration, and functional exhaustion within the tumor microenvironment. We also highlighted recent advances in antigen discovery, TCR/CAR engineering, and strategies to enhance T cell persistence and cytotoxicity. These insights underscore the complex interplay between tumor biology and therapeutic efficacy, emphasizing the need for innovative strategies to overcome current limitations.

**Figure 5:**
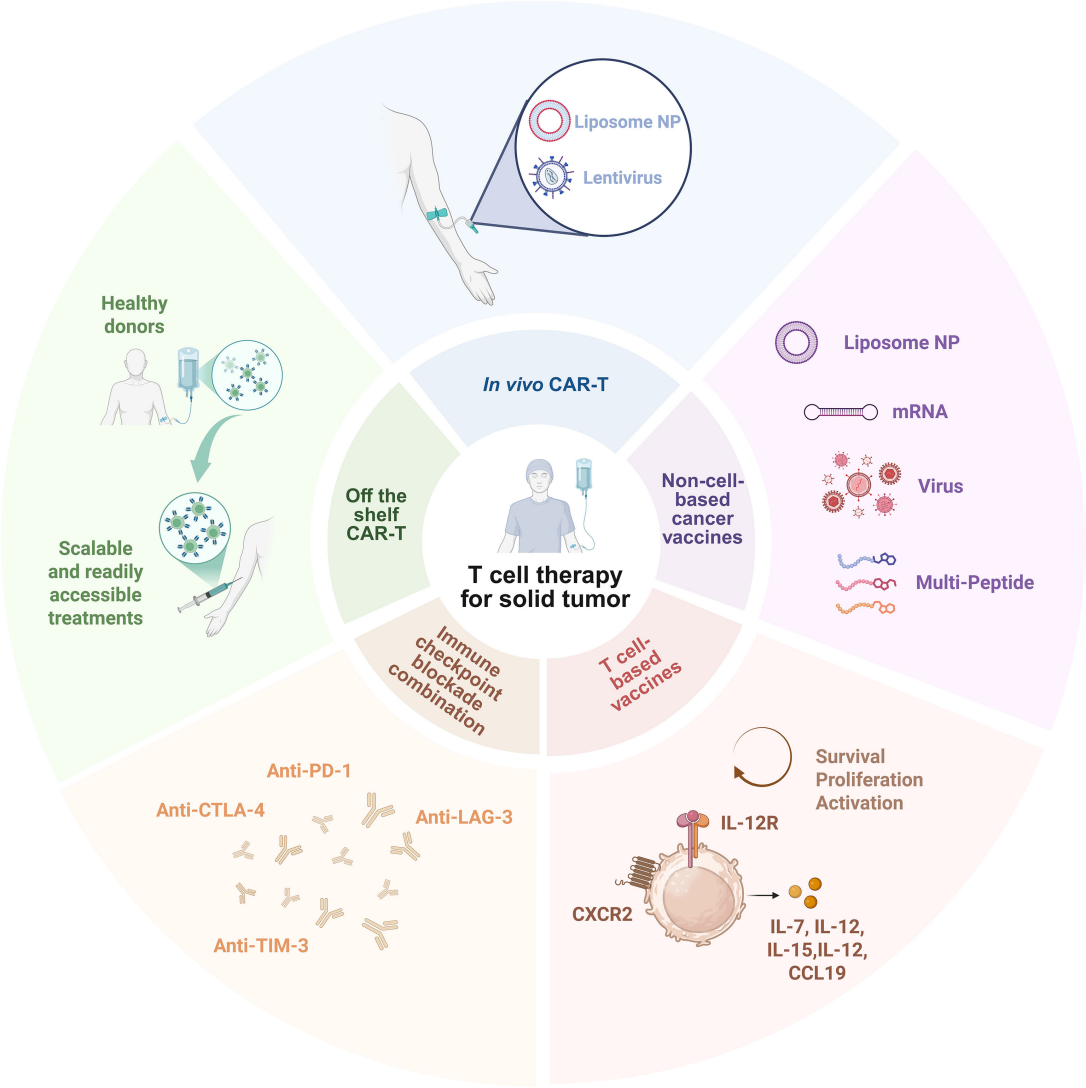
Overview of representative T cell–based therapeutic strategies for solid tumors. The inner circle summarizes the emerging strategies to enhance T cell–based immunotherapy for solid tumors, including *in vivo* CAR-T approaches, off-the-shelf CAR-T strategies, non–cell-based cancer vaccines, T cell-based vaccines and immune checkpoint blockade. The outer circle highlights the specific delivery platforms and engineering approaches used to implement these strategies. This figure was created in BioRender.

**Figure 5** further illustrates the diverse key strategies being developed to address these challenges and improve the efficacy of T cell-based therapies in solid tumors. These include: *in vivo* CAR-T approaches that deliver CAR constructs directly into endogenous T cells using lentiviral vectors or lipid nanoparticles; off-the-shelf CAR-T therapies that utilize allogeneic T cells derived from healthy donors for scalable and readily accessible treatments; non–cell-based cancer vaccines such as mRNA, viral vector, and multi-peptide platforms designed to stimulate tumor-specific T cell responses; T cell–based vaccines engineered to express survival and activation signals (e.g., IL-12, CXCR2) that enhance T cell persistence and function within the tumor microenvironment; and combination therapies involving immune checkpoint blockade (e.g., anti–PD-1, anti-CTLA-4, anti-LAG-3, anti-TIM-3) to reinvigorate exhausted T cells and amplify therapeutic responses. Together, these strategies aim to overcome the immunosuppressive barriers of solid tumors and expand the clinical applicability of T cell immunotherapy.

Despite the promising potential of non–cell-based cancer vaccines in stimulating tumor-specific T cell responses, they still face several inherent limitations that restrict their clinical efficacy in solid tumor immunotherapy. First, antigen delivery efficiency is suboptimal: most non–cell-based vaccines (e.g., mRNA, peptide vaccines) lack specific targeting to professional antigen-presenting cells (APCs) such as dendritic cells (DCs), leading to limited antigen uptake and presentation, which weakens the subsequent T cell activation. Second, their immunogenicity is often insufficient, especially in patients with advanced solid tumors who have an immunosuppressive tumor microenvironment (TME) and compromised immune systems; standalone non–cell-based vaccines are usually unable to induce robust and durable anti-tumor immune responses, requiring combination with adjuvants or other immunotherapies (e.g., checkpoint inhibitors) to enhance efficacy. Third, therapeutic responses are highly heterogeneous due to individual differences in HLA genotypes and immune status: the same vaccine may elicit strong immune responses in some patients but no response in others, as HLA molecules exhibit high polymorphism and only bind to specific antigen epitopes. Fourth, there is a risk of off-target immune responses or immune tolerance: if the vaccine targets tumor-associated antigens (TAAs) that are also weakly expressed in normal tissues, it may induce autoimmunity; conversely, prolonged exposure to low-immunogenicity antigens may lead to immune tolerance, reducing the anti-tumor effect. Fifth, the development of personalized neoantigen-based non–cell-based vaccines faces technical and cost barriers, including the complexity and high cost of neoantigen prediction, validation, and personalized synthesis, which limits their widespread clinical application.

Synthesizing the core content of this review, the advances in T cell-based immunotherapies for solid tumors exhibit distinct advantages while still facing non-negligible challenges. In terms of advantages, the precise identification of tumor-specific antigens (such as neoantigens) has laid a foundation for targeted T cell therapy, significantly improving the specificity of treatment and reducing off-target toxicities. Meanwhile, the engineering optimization of TCR/CAR constructs (including affinity maturation, co-stimulatory molecule modification, and logic-gate design) has effectively enhanced the anti-tumor activity of T cells and reduced the risk of cytokine release syndrome (CRS). Additionally, strategies targeting the tumor microenvironment (such as checkpoint blockade, cytokine therapy, and depletion of immunosuppressive cells) have broken the immune suppression state in solid tumors, promoting T cell infiltration and functional persistence, thereby improving the therapeutic response rate in solid tumor patients.

However, these therapeutic strategies still have obvious limitations. For tumor antigen heterogeneity, although neoantigen screening technologies have made progress, the high variability of neoantigens among different patients and even within the same patient’s tumor tissue still leads to incomplete tumor elimination and increased risk of recurrence. In terms of TCR/CAR engineering, despite the optimization of constructs, the occurrence of on-target off-tumor toxicity (especially for antigens expressed in normal tissues at low levels) and the exhaustion of engineered T cells during long-term anti-tumor responses remain important factors restricting therapeutic efficacy. For strategies targeting the tumor microenvironment, the complexity and plasticity of the tumor microenvironment lead to inconsistent therapeutic responses among patients; moreover, the combined use of multiple agents may increase the risk of adverse reactions, requiring further optimization of combination regimens and dosages.

Based on the above analysis, the future outlook of T cell-based immunotherapies for solid tumors should focus on the following directions: First, developing more efficient and comprehensive tumor antigen screening and verification technologies, such as multi-omics integrated analysis and *in vitro* high-throughput antigen validation platforms, to address the challenge of tumor antigen heterogeneity. Second, optimizing TCR/CAR engineering strategies, including the development of universal TCR/CAR frameworks with better safety profiles, the exploration of novel co-stimulatory signaling pathways to delay T cell exhaustion, and the combination of gene editing technologies (such as CRISPR-Cas9) to knock out inhibitory molecules in T cells. Third, conducting in-depth studies on the mechanism of tumor microenvironment regulation, identifying new key regulatory targets, and developing personalized combined therapeutic regimens based on the individual characteristics of patients’ tumor microenvironment to improve the universality and effectiveness of treatment. Fourth, establishing more accurate preclinical models (such as patient-derived xenograft models and organoid models) that can simulate the biological characteristics of human solid tumors, to better evaluate the efficacy and safety of novel therapeutic strategies and accelerate their translation to clinical applications.

Notably, transformative approaches represented by *in vivo* T cell engineering and universal off-the-shelf T cell products, which are highlighted as two key strategies in **Figure 5**, emerge as important supplements to traditional therapeutic directions and may provide new solutions for overcoming current limitations. These approaches are now driving T cell-based therapies into a new era of development, with substantial progress in preclinical and clinical research.

For *in vivo* T cell engineering, reprogramming of endogenous T cells using viral vectors or lipid nanoparticles enables direct delivery of CAR or TCR constructs, circumventing the logistical and manufacturing burdens of ex vivo processes while improving scalability, accessibility, and cost-effectiveness. Recent studies have demonstrated the feasibility and therapeutic potential of this strategy. For instance, ESO-T01, a lentiviral vector targeting BCMA, showed promising safety and efficacy in patients with relapsed/refractory multiple myeloma, eliminating the need for leukapheresis and intensive preconditioning (193). Another approach, INT2104, employs membrane-bound targeting domains and de-targeted fusogens to direct CAR transgenes into CD7^+^ T and NK cells *in vivo*, generating functional CAR-T and CAR-NK cells after a single injection without lymphodepletion (194). Lipid nanoparticle-based systems have also been developed to deliver mRNA encoding CARs into specific T cell subsets, demonstrating tumor control in humanized mouse models and immune resetting in nonhuman primates (195). Mechanistic insights into the transduction process, such as the role of ERK pathway activation—are helping optimize *in vivo* CAR-T cell conversion efficiency (196). As summarized by recent reviews and news reports, these platforms are entering clinical trials with the potential to lower treatment costs, broaden accessibility, and expand therapeutic indications beyond cancer to autoimmune diseases (197, 198).

Concurrently, allogeneic “off-the-shelf” CAR-T therapies are gaining traction as a scalable and readily accessible alternative. By genetically modifying healthy donor T cells, universal CAR-T products can be manufactured in bulk and stored for on-demand administration, reducing variability and ensuring rapid treatment delivery. Several early-phase clinical trials have reported encouraging outcomes. Cemacabtagene ansegedleucel (cema-cel), an anti-CD19 allogeneic CAR-T product, achieved an overall response rate (ORR) of 58% and a complete response (CR) rate of 42% in patients with relapsed/refractory large B-cell lymphoma, with a median CR duration of 23.1 months (199). UCART19, a genome-edited donor-derived product, induced CR/CRi in 67% of pediatric and adult patients with B-ALL within 28 days post-infusion (200). Similarly, BCMA-targeting ALLO-715 and CD7-targeting GC027 have demonstrated high response rates of 70.8% and 91.7% respectively in patients with multiple myeloma and T-ALL (201, 202).

Nevertheless, the application of these transformative approaches in solid tumors still needs to address specific challenges. For *in vivo* engineering, efficient *in vivo* targeting of T cells in the tumor microenvironment remains a key hurdle; for allogeneic off-the-shelf therapies, the risk of graft-versus-host disease (GVHD) and immune rejection requires further mitigation. Future research should focus on resolving these solid tumor-specific obstacles to fully leverage the potential of these innovative strategies.

Together, these technological advances reflect a paradigm shift in the design, delivery, and deployment of T cell immunotherapies. *In vivo* engineering and universal T cell platforms offer solutions to scalability, cost, and accessibility challenges, while retaining or even enhancing therapeutic efficacy. Despite ongoing challenges such as immunogenicity, graft-versus-host disease (GVHD), and risks associated with gene editing, both *in vivo* and off-the-shelf CAR-T strategies represent crucial innovations in the next generation of cellular immunotherapy. Together as summarized in **Graphical Abstract**, they offer the promise of more accessible, affordable, and broadly applicable treatments for cancer and beyond.

**Figure 5** also illustrates key strategies being developed to improve the efficacy of T cell-based therapies in solid tumors. These include *in vivo* CAR-T approaches that deliver CAR constructs directly into endogenous T cells using lentiviral vectors or lipid nanoparticles; off-the-shelf CAR-T therapies that utilize allogeneic T cells derived from healthy donors for scalable and readily accessible treatments; non–cell-based cancer vaccines such as mRNA, viral vector, and multi-peptide platforms designed to stimulate tumor-specific T cell responses; T cell–based vaccines engineered to express survival and activation signals (e.g., IL-12, CXCR2) that enhance T cell persistence and function within the tumor microenvironment; and combination therapies involving immune checkpoint blockade (e.g., anti–PD-1, anti-CTLA-4, anti-LAG-3, anti-TIM-3) to reinvigorate exhausted T cells and amplify therapeutic responses. Together, these strategies aim to overcome the immunosuppressive barriers of solid tumors and expand the clinical applicability of T cell immunotherapy.
